# Intraocular deposits and cataracts after long-term rifabutin intake: A case report

**DOI:** 10.1097/MD.0000000000020049

**Published:** 2020-05-22

**Authors:** Kohei Harada, Masafumi Uematsu, Ryotaro Ueki, Mao Kusano, Yoshihisa Yamada, Yasser Helmy Mohamed, Takashi Kitaoka

**Affiliations:** Department of Ophthalmology and Visual Sciences, Graduate School of Biomedical Sciences, Nagasaki University, Japan.

**Keywords:** cataracts, corneal endothelial deposits, intraocular deposits, iridocorneal angle deposits, rifabutin

## Abstract

**Rationale:**

Rifabutin is a broad-spectrum antibiotic known to cause deposits on the corneal endothelium and lens. We report a patient in whom cataracts developed and progressive pigment deposits were seen on the corneal endothelium, lens, and iridocorneal angle.

**Patient concerns:**

The patient was a 45-year-old woman who had been received long-term treatment with a combination of various anti-mycobacterial drugs for multidrug-resistant tuberculosis starting in 2004. Rifabutin was started in 2009, and she was referred to our department in 2017 for detailed ophthalmological examination.

**Diagnoses:**

Both eyes showed pigmented deposits over the entire corneal endothelium, the entire periphery of the iridocorneal angle, and the anterior surface of the lens. Mild cataracts were also diagnosed bilaterally. Pigment deposits on the anterior surface of the lens and the cataracts in both eyes gradually progressed. These lesions were assumed to be associated with long term rifabutin intake.

**Interventions:**

Rifabutin intake was discontinued after progression of intraocular deposits, cataracts, and ERG deterioration.

**Outcomes:**

Visual acuity improved, although cataracts, deposits, and ERG deterioration remained.

**Lessons:**

Rifabutin may induce not only corneal endothelial deposits, but also cataracts and iridocorneal angle deposits.

## 1 Introduction

Rifabutin is a broad-spectrum antibiotic which is used not only for severe infections such as tuberculosis and osteomyelitis, but also as one part of treatment for Mycobacterium avium complex (MAC), a lung disease common in patients with acquired immunodeficiency syndrome.^[1]^ Known side effects of rifabutin include drug-induced uveitis with subsequent intraocular deposits, and drug deposits on the corneal endothelium and the anterior surface of the lens. Our case report describing a patient treated with rifabutin in whom iridocorneal angle pigment deposits and cataracts were diagnosed in addition to deposits on the corneal endothelium and anterior surface of the lens. This case report was approved by the ethical committee of Nagasaki University Hospital, and the patient has provided informed consent for publication.

## 2 Case

Our patient is a 45-year-old woman with past history of left pneumonectomy in 2014 for multi-drug resistant tuberculosis. The patient was treated for long-term with a combination of various anti-mycobacterial drugs (enviomycin, cycloserine, ethionamide, clarithromycin, sitafloxacin, others) for multi-drug resistant tuberculosis starting in 2004. In 2009, she started treatment with oral rifabutin 150 to 300 mg/day (3–6 mg/kg/day) periodically with total intake period of 95 months. Repeated episodes of remission and exacerbation were experienced, and she was admitted to the Department of Infectious Diseases in our hospital in February 2017 to undergo multidrug therapy with potassium clavulanate/amoxicillin, cycloserine, linezolid, delamanid, clofazimine, rifabutin, meropenem, and enviomycin for refractory multidrug-resistant Mycobacterium tuberculosis. She was referred to the Department of Ophthalmology for a careful examination of drug side effects.

**Figure 1:**
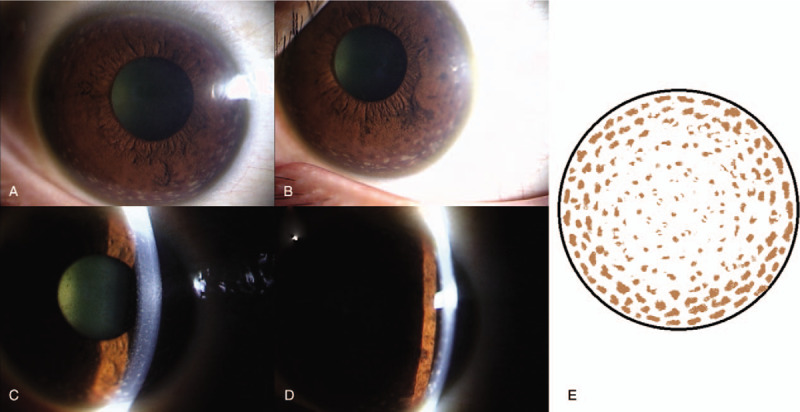
Pigment deposits are seen concentrically on the entire corneal endothelium of both eyes, with decreasing density toward the center of the cornea. (a, c) Anterior segment photo (right eye) at initial examination. (b, d) Anterior segment photo (left eye) at initial examination. (e) Cornea schema at initial examination.

**Figure 2:**
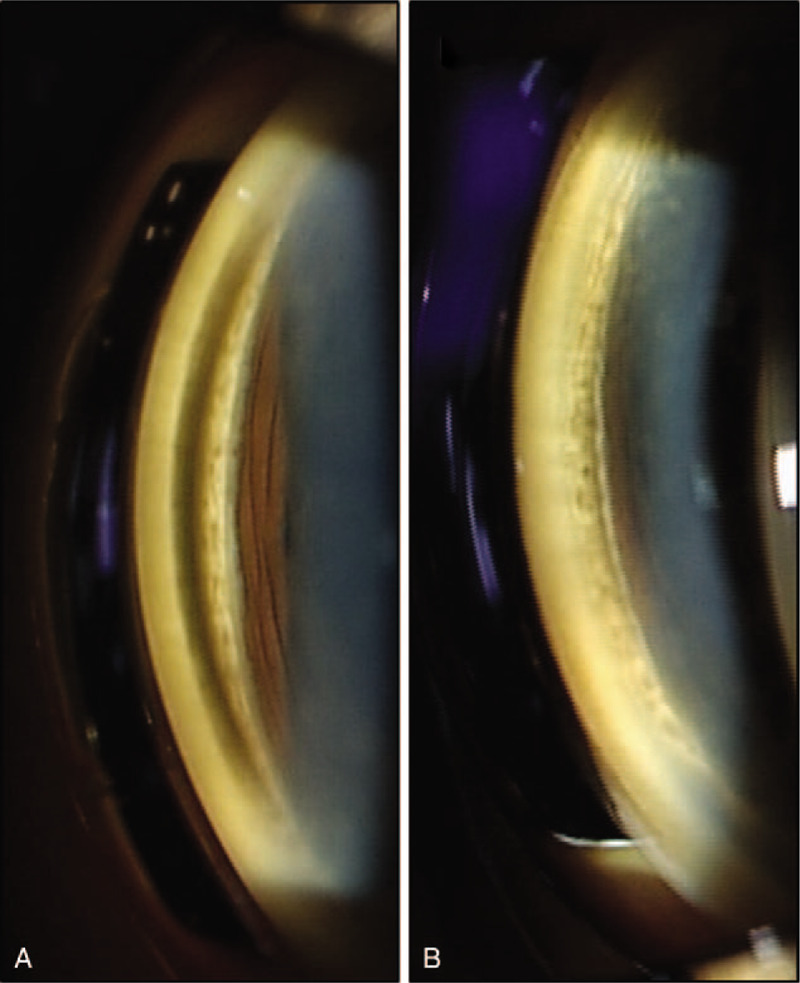
Pigment deposits are seen on the entire periphery of the iridocorneal angle. (a) Right eye on initial examination. (b) Left eye on initial examination.

**Figure 3:**
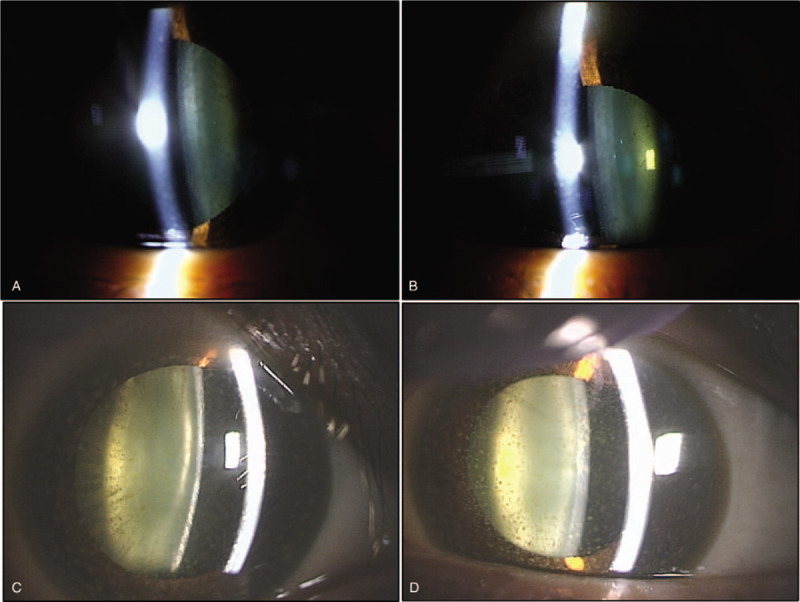
Increased pigment deposits and cataract progression are seen on the anterior surface of the lens of both eyes. (a) Right eye on initial examination. (b) Left eye on initial examination. (c) Right eye after 17 months. (d) Left eye after 17 months.

**Figure 4:**
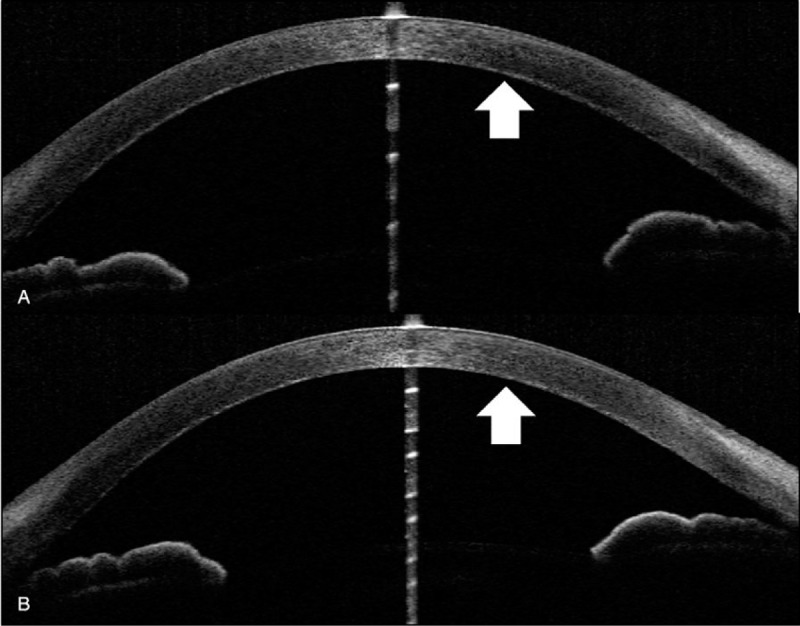
Findings of high-brightness deposits on the Descemet membrane (white arrows) of both eyes. (a) Anterior segment OCT of right eye on initial examination. (b) Anterior segment OCT of left eye on initial examination. OCT: optical coherence tomography.

Initial findings: Visual acuity was 0.3 (uncorrected visual acuity) (best corrected visual acuity = 0.8) in the right eye and 0.3 (0.9) in the left eye, and intraocular pressure was 15 mm Hg and 14 mm Hg in the right and left eye respectively. The median critical flicker fusion frequency was 35.2 Hz in the right eye and 34.3 Hz in the left eye, showing no abnormality. No abnormalities were evident on the Standard Pseudoisochromatic Plates–Part 2 (SPP-2) for acquired abnormalities. Yellowish brown pigment deposits were distributed all over the entire body of the patient skin, including the face. Yellowish-brown pigment deposits were seen concentrically on the entire corneal endothelium of both eyes, with decreasing density toward the center (Fig. 1a–e). The iridocorneal angle was open, with presence of pigment deposits without dandruff-like material (Fig. 2a, b). No signs of inflammation including cells and flare in the anterior chamber were noticed. Iris was intact with normal pigmentation seen in Japanese population without mid peripheral trans-illumination, nor deposition of flakes related to exfoliation syndrome. Both lenses showed pigment deposits on the anterior surface and mild cataracts were diagnosed without visible 3 ring sign of exfoliation syndrome (Fig. 3a, b). With optical coherence tomography (OCT) of the anterior segment of the eye, deposits of high-brightness were seen on the Descemet membrane of both eyes (Fig. 4a, b). Corneal endothelial cell density was 3105 cells/mm^2^ in the right eye and 3112 cells/mm^2^ in the left eye. No abnormalities were diagnosed in the retina on fundus examination or OCT, while mildly decreased sensitivity was seen around Mariottes blind spot in the left eye on a dynamic visual field test.

Progress: Regular follow-up examinations were made every 1 to 2 months. After 17 months, increased pigment deposits were detected on the anterior surface of the lens in both eyes with progression of cataracts (Fig. 3c, d). Visual acuity had decreased to 0.15 (0.2) in the right eye and 0.1 (0.4) in the left eye. On the SPP-2, 5 plates were misread with the right eye and 6 with the left eye, indicating mild abnormality, but no abnormalities were evident in median critical flicker fusion frequency, which was 31.5 Hz in the right eye and 30.0 Hz in the left eye. Other tests showed no marked changes including fundoscopy, visual field test, fluorescein fundus angiography, and corneal endothelial cell density. With flash electroretinogram (ERG) test, negative ERG with smaller b-waves than a-waves were seen in both eyes. Two weeks later, the condition had deteriorated, with 11 plates misread in the right eye and 6 in the left eye on SPP-2 and median critical flicker fusion frequency was 29.8 Hz in the right eye and 27.7 Hz in the left eye. Decreased visual acuity was assumed to be due to worsening pigmentation of the cornea and retina, cataract progression, drug-induced retinal disorder, and drug-induced neuropathy. Two days later, the medical treatment for tuberculosis was completed, and all anti-mycobacterial drugs were discontinued. Seven months after drug discontinuation, test results improved to no error in both eye on the SPP-2, median critical flicker fusion frequency was 37.3 Hz in the right eye and 38.5 Hz in the left eye, and visual acuity was 0.2 (0.6) in the right eye and 0.2 (0.8) in the left eye. There is no improvement of negative-type ERG, and the status of deposits and cataracts were maintained.

## 3 Discussion

There were no signs of diseases which present corneal endothelial deposits such as uveitis, herpetic endotheliitis, pigment dispersion syndrome, iridocorneal endothelial syndrome, Fuchs dystrophy, exfoliation syndrome, and others. Because the patient presented with skin pigmentation, we speculated generalized drug deposition in whole body including eyes. Drugs with reported involvement in corneal deposits are shown in Table 1.^[2,3]^ Among the patients drug, only rifabutin and clofazimine cause corneal deposits. Because deposits were seen in the corneal endothelium of the present case, it were attributed to rifabutin. Pigment deposits due to clofazimine are crystalline and present mainly in the corneal epithelium, and thus can be ruled out in the present case.^[4,5]^

**Table 1:**
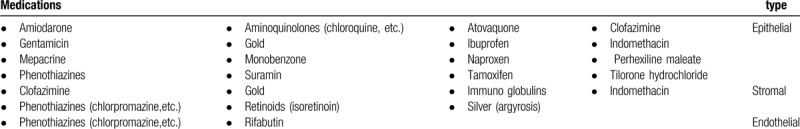
Drug deposits on the cornea (cited from Barlett et al^[2]^ and Hollander et al^[3]^).

Rifabutin is a member of the rifamycin family of drugs, and is used to treat MAC, tuberculosis, and atypical mycobacteriosis. Rifabutin has two mechanisms which may lead to the intraocular deposits. The first through deposits accompanying drug-induced uveitis and the second through deposits of the drug itself. In drug-induced uveitis, findings similar to acute anterior uveitis are seen, such as ciliary hyperemia, fibrin deposition, hypopyon, posterior synechia, and intraocular deposits as a result of inflammatory cell aggregation.^[6]^ In contrast, deposits accompanying the drug itself develop without inflammation and characterized by its yellowish brown color which distributed concentrically in a stellate or reticulate shape on the corneal endothelium. The corneal endothelial deposits are characterized by higher density and larger size on the periphery of the cornea, while the density at the center is lower and the shape is also finer.^[7]^ In the present case, pigment deposits are thought to be associated with the drug itself due to absence of intraocular inflammation and the shape and distribution of pigment deposits. In a previous case report, corneal deposit appeared after 6 months of 300 mg/day rifabutin intake (Total intake is approximately 54 g).^[7]^ In other case series of children, dosage and period of rifabutin intake were 5 to 15 mg/day, and 25 to 43 months, respectively.^[8]^ Rifabutin intake in our case (3 or 6 mg/kg/day in 95 months with total intake amount of 697 g) was more than the previous cases.

Rifabutin pigment deposits are seen not only in the skin and eyes, but also in organs of the entire body.^[9]^ Deposits in the eye include corneal Descemet membrane and the anterior surface of the lens.^[10]^ This is the first report in which deposits on the Descemet membrane could be confirmed with anterior segment OCT. Ponjavic et al, reported the side effects of rifabutin on the retinal tissue and cone-and-rod dysfunction on ERG as seen in the present case.^[7]^ Rifabutin deposits are thought to occur due to high lipid solubility, protein-binding, pH, and ion interactions of rifabutin.^[8]^ The specific composition of deposits is not known, but Brughera et al in a toxicological study of rats, confirmed that the deposits that occur in organs throughout the entire body are lipofuscin-like substances.^[9]^ Myers et al showed that cataracts occurred with overdoses of rifabutin in an animal experiment,^[11]^ but no clinical reports in humans have been described. This case may be the first human report describing cataract as a complication to long-term rifabutin intake. Although no reports have described pigment deposits in the iridocorneal angle, the present case on long-term rifabutin was assumed to be the cause.

It is reported that although ERG abnormalities and pigment deposits of skin may improve after discontinuation of the rifabutin, but corneal endothelial deposits are irreversible and maybe even get worsen.^[7]^ Careful ophthalmological examination including cornea, iridocorneal angle, lens, and retinal function must be done in cases with long term rifabutin intake.

## Author contributions

**Conceptualization:** Masafumi Uematsu.

**Investigation:** Ryotaro Ueki, Mao Kusano, Yoshihisa Yamada.

**Supervision:** Takashi Kitaoka.

**Writing – original draft:** Kohei Harada.

**Writing – review & editing:** Masafumi Uematsu, Yasser Helmy Mohamed.
